# Enhancement of Polyphenols and Antioxidant Activity in Germinated Black Highland Barley by Ultrasonication

**DOI:** 10.3390/molecules28093679

**Published:** 2023-04-24

**Authors:** Jie Zhang, Junling Guo, Bin Dang, Wengang Zhang, Wancai Zheng, Xijuan Yang

**Affiliations:** 1 Academy of Agriculture and Forestry Sciences, Qinghai University, Xining 810016, China; zjzj89zjzj@163.com (J.Z.); zhangwgang0402@sina.com (W.Z.); 13565849218@163.com (W.Z.); 2 Qinghai Tibetan Plateau Key Laboratory of Agricultural Product Processing, Qinghai Academy of Agriculture and Forestry Sciences, Xining 810016, China; 18397101505@163.com; 3 Laboratory for Research and Utilization of Qinghai Tibet Plateau Germplasm Resources, Qinghai Academy of Agriculture and Forestry Sciences, Xining 810016, China

**Keywords:** black highland barley, polyphenols, ultrasound, germination, antioxidant activity

## Abstract

The aim of this study was to investigate the effect of ultrasonic stress germination (USG) on total phenolic contents (TPC), total flavonoid contents (TFC), the phenolic compositions, and antioxidant activities of black highland barley (BHB). The USG processing parameters, polyphenol profile, phenolic compositions, and antioxidant activities were explored after USG. Results showed that the optimal USG parameters were as follows: 350 W ultrasonic pretreatment power, 30 °C ultrasonication temperature, 25 min ultrasonication time, and 64 h germination time. Under these conditions, the total phenolic content (688.84 ± 5.30 mg/100 g) and total flavonoid content (59.23 ± 0.45 mg/100 g) of BHB were increased by 28.55% and 10.15%, respectively, compared to the untreated samples. In addition, the USG treatment could more effectively enrich bound phenolic acids and free flavonoids, among which the content of catechin was significantly increased by USG and was the main characteristic substance. Moreover, the USG treatment could improve the antioxidant activity and had a higher antioxidant potency composite index (APC index) (97.91%) of BHB. These results indicate that USG might be an effective method to enrich polyphenols and improve antioxidant activity in BHB.

## 1. Introduction

Highland barley is a special crop grown on the Tibetan plateau, which is mainly distributed in alpine regions at altitudes of 4200~4500 m. The crop is characterized by cold tolerance, early maturity, wide adaptability, and stable yield [1]. Black highland barley (BHB), a valuable germplasm resource [2], is rich in β-glucan, dietary fibers, resistant starch, and polyphenols [3,4], with glucose- and lipid-lowering, anticancer, and antioxidant properties [5]. Recent studies and applications of active substances in barley have focused on β-glucan, dietary fibers, and polyphenols. Polyphenols have an aromatic structure, including either one or more hydroxyl groups and which were classified into different subclasses based upon the number of phenol ring systems that they contain, saturation, and length of the carbon chain that bind the rings to one another. They contain both small molecular weight phenolic acids and large molecular weight tannins. A large number of studies have confirmed polyphenols have good antioxidant, anticancer, anti-inflammatory, and antibacterial activities. In addition, polyphenols and their associated metabolites affect intestinal health and the balance of intestinal microbiota by stimulating the growth of beneficial bacteria and inhibiting the proliferation of pathogens [6]. Several studies have shown that phenolic resources are abundant in colored barley and show high bioactivity [7]. However, polyphenols exist primarily in the bound form in cereals and are mainly (about 95%) bound to the cell wall polysaccharides [8]. Therefore, only a few compounds effectively exert their functional activity; owing to low bioavailability, exploiting their value in practical applications remains difficult. Therefore, to improve the utilization of cereal polyphenols, enrichment techniques to enhance the content and bioactivity of cereal polyphenols are effective. This may have implications for the development and utilization of BHB and its phenolic substances.

Germination is an effective way to improve and enhance the tissue structure, nutritional properties, and functional characteristics of plants [9,10]. It enhances the enzymatic activities of α-amylase and protease in grains and increases the content of total phenolic content and antioxidant activity, such as that reported in chickpea [11], buckwheat [12], and oat [13]. Epidemiological studies have revealed that germinated whole grains have antidiabetic, antihypercholesterolemic, and anticancer properties and can also improve the intestinal microbiota [14,15].

To efficiently exploit the advantages of germination technology and improve the biological activity of whole grains, germination pretreatment of whole grains has been emphasized in recent years. Whole grain pretreatment is primarily based on ultrasonic, soaking, high hydrostatic pressure, enzyme, and low-temperature plasma treatment [16]. Different pre-germination treatments have differential effects on the quality of whole grains. Non-thermal processing pretreatment exerts positive effects on organoleptic, nutritional, and processing qualities of germinated whole grains relative to normal soaking [17,18]. However, high levels of chemical and metal ion stress can lead to residual metal ions in grain and contaminate the environment. The cold and heat shock stress, mechanical damage, and drought stress cause irreversible damage to grain [19].

Ultrasonic treatment, a safe and efficient physical stimulation, not only enhances the nutritional value of raw materials but also maximizes the flavor retention of raw materials, and therefore is now widely used for the pretreatment of seeds and grains, including soybeans [20], brown rice [21], wheat [22], and red rice [23], to increase the nutritional value and secondary metabolites, such as GABA and phenolic compounds, in germinating seeds and shoots. Ding shows that ultrasonic treatment for 5 min significantly increases the content of total phenolic, avenanthramides, and γ-aminobutyric acid in germinated oat [24]. A previous study on sorghum shoots treated with sonication (sonication at 40% amplitude for 5 min) showed superior phytochemical composition, radical scavenging activity and phenolic profile after germination [25]. The current study on the effects of germination on the nutritional and functional components of barley only reported the differences in basic nutrients and polyphenol composition of highland barley under normal germination conditions [26] and the improvement of functional nutrients, sensory quality, and physicochemical properties of highland barley after germination under anoxic stress [19]. In this study, ultrasonic pretreatment was chosen to improve the polyphenol of sprouted BHB, because of the limited efficiencies of sprouting in inducing biotransformation [27] and the high cost of anoxic stress. However, little is known about the effect of ultrasonic stimulation on the polyphenols of germinated BHB. In addition, it is reported that excessive ultrasonic treatment can cause damage to seeds, resulting in decreased degradation of phenolic substances [28].

This study aimed to explore an optimum USG condition of BHB in order to obtain germinated BHB with high nutritional value. Meanwhile, total phenolic content (TPC), total flavonoid content (TFC), the phenolic compositions (phenolic acid, flavonoids, and monomeric phenols) and antioxidant activities (DPPH· scavenging ability, FRAP reducing power, and ABTS^+^· scavenging ability) were also evaluated. The findings may provide an economic, green, and efficient method for the production of polyphenols- enriched functional food and improve the utilization of BHB.

## 2. Results and Discussion

### 2.1. Response Surface Test

The Box–Behnken RSM was used to optimize the processing parameters for enriching polyphenols in BHB by USG based on TPC (Y_1_) and TFC (Y_2_) as response values. The independent variables were germination time (X_1_), ultrasound power (X_2_), and ultrasound time (X_3_). The results are shown in Table 1 and Table 2. 

Fitting was performed by quadratic multiple regression using the Design Expert 10.0.1 software to obtain the following equations for TPC (Y_1_) and TFC (Y_2_), correspondingly, for the independent variables X_1_, X_2_, and X_3_, respectively:

Y_1_ (TPC) = 681.22 + 23.63 X_1_ + 9.44 X_2_ − 7.33 X_3_ + 7.23 X_1_ X_2_ + 5.23 X_1_ X_3_ − 6.65 X_2_ X_3_ − 35.03 X_1_^2^ − 47.97 X_2_^2^ − 29.11 X_3_^2^

Y_2_ (TFC) = 58.85 + 5.13 X_1_ + 0.27 X_2_ − 1.13 X_3_ − 0.43 X_1_ X_2_ + 0.80 X_1_ X_3_ − 0.39 X_2_ X_3_ − 4.51 X_1_^2^ − 2.53 X_2_^2^ − 1.71 X_3_^2^

The results of the analysis of variance in Table 2 suggest that the fitted models for TPC and TFC were both highly significant (*p* < 0.0001), while the lack of fit was not significant (*p*-values: 0.2161 and 0.3343, respectively), indicating a good fitness for the models. Thus, the above regression equations could be used to analyze the experimental results instead of the experimental true points. The correlation coefficient, R^2^, of the two models was 0.9755 and 0.9978, respectively, and the adjustment coefficient, R^2^_adj_, was 0.9441 and 0.9950, respectively, indicating a good fit between the predicted and actual values. Therefore, the model could well characterize the dynamic relationship between the variables and the response values. Therefore, the model could be used to analyze and predict the optimal process parameters for enriching TPC and TFC from BHB by USG.

**Figure 1:**
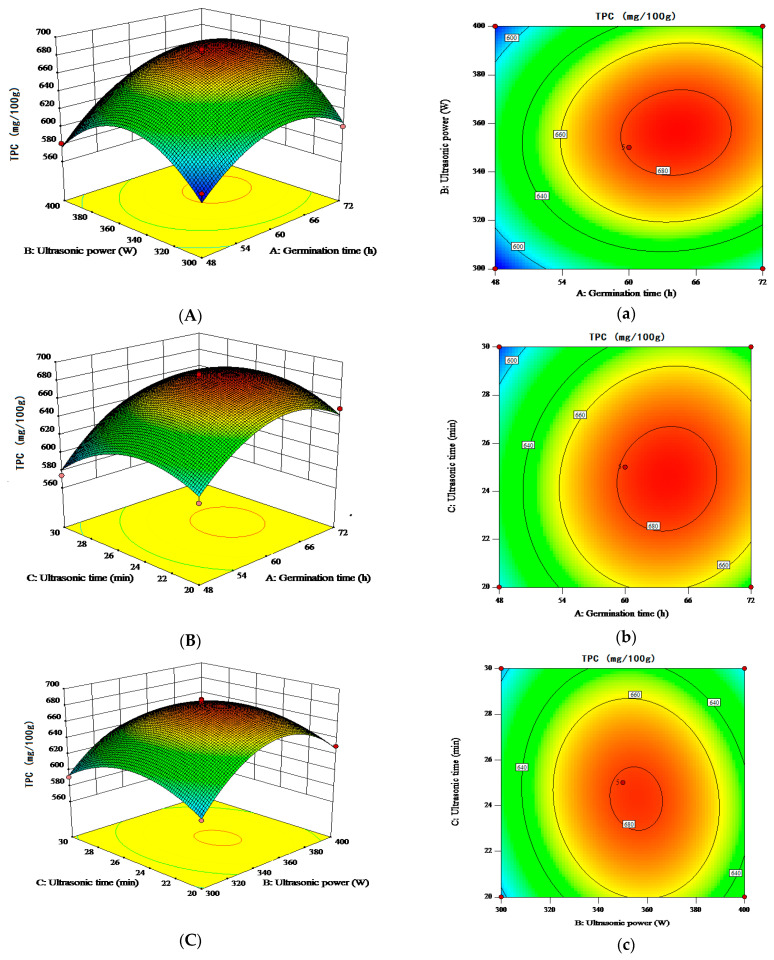
Response surface and contour maps of the interactions between two factors on TPC. (**A**,**a**) germination time and ultrasound power; (**B**,**b**) germination time and ultrasound time; (**C**,**c**) ultrasound power and ultrasound time.

**Figure 2:**
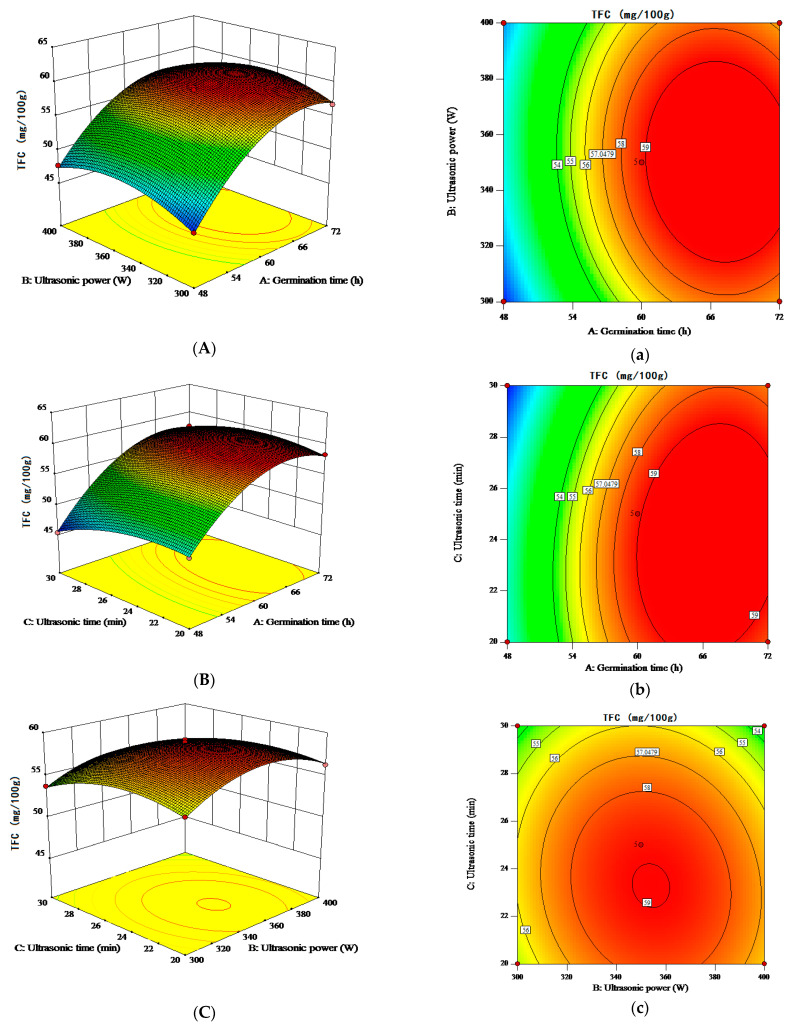
Response surface and contour maps of the interactions between two factors on TFC. (**A**,**a**) germination time and ultrasound power; (**B**,**b**) germination time and ultrasound time; (**C**,**c**) ultrasound power and ultrasound time.

**Figure 3:**
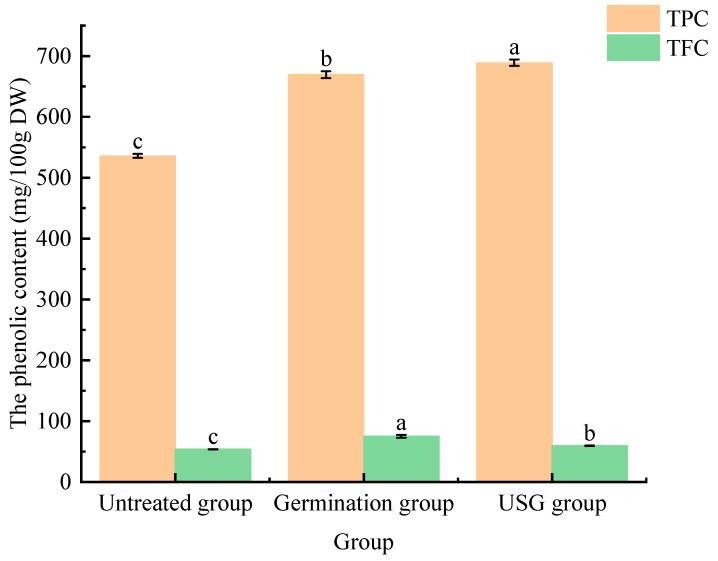
The TPC and TFC in different treatment of BHB. Lower-case letters in the figure respectively indicate significant differences among different treatment of BHB about TPC and TFC (*p* < 0.05).

The response surface test plots and contour plots for different factors on TPC and TFC are shown in Figure 1 and Figure 2. As shown in Figure 1, the contour lines of the interactions are all circular and these are not significant. As shown in Figure 2, the contours lines of the interaction terms X_1_X_3_ and X_1_X_2_ are elliptical and there is a maxima for each response value. When ultrasonic pretreatment power was 356.57 W for 24.45 min, and germination time was 64.11 h, the predicted value of TPC was 686.30 mg/100 g. When ultrasonic pretreatment power was 351.11 W for 23.97 min, and germination time was 66.58 h, the predicted value of TFC was 60.38 mg/100 g. The predicted values were validated based on the optimal process parameters to examine the reliability of these results. However, considering the limitation of the instrument and energy consumption, the optimal process parameters were adjusted to 64 h germination time, 350 W ultrasound power, and 25 min ultrasound time. Three parallel tests were conducted under these conditions. The average TPC and TFC of the USG sample were 688.84 ± 5.30 mg/100 g and 59.23 ± 0.45 mg/100 g, respectively, which were significantly higher than those in the untreated sample (535.84 mg/100 g and 53.77 mg/100 g, respectively). In addition, USG treatment was able to significantly increase the TPC compared to the germination treatment (Figure 3). The actual values of TPC and TFC obtained from parallel tests conducted under the optimal conditions predicted using the regression model were close to those predicted by the model. Therefore, the obtained regression model indicates the feasibility of process optimization and may have implications for its utility.

### 2.2. Effects of USG on the Phenolic Compounds in BHB

**Figure 4:**
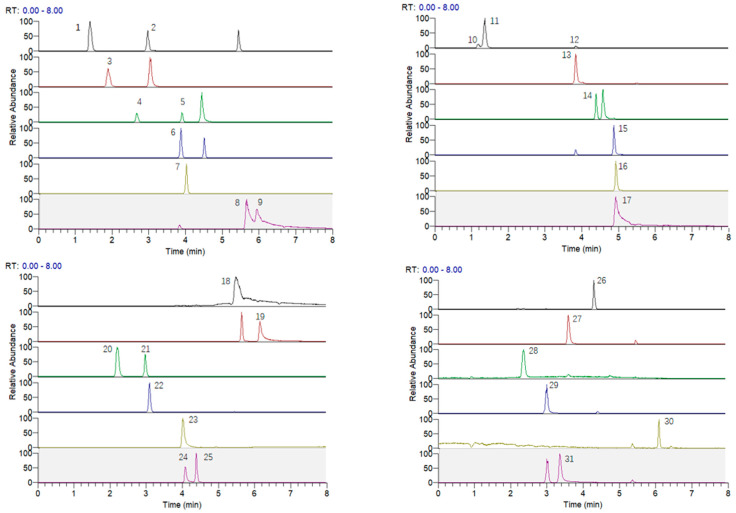
Extraction ion current chromatogram for 31 phenolic compound standard samples. 1: homogentisic acid; 2: vanillic acid; 3: protocatechuic acid; 4: *p*-hydroxybenzoic acid; 5: sesamol; 6: *p*-coumaric acid; 7: ferulic acid; 8: kaempferol; 9: luteolin; 10: phlorogucinol; 11: pyrogallol; 12: maltol; 13: taxifolin; 14: rutin; 15: diosmin; 16: kaempferol-3-O-rutinoside; 17: myricetin; 18: quercetin; 19: diosmetin; 20: catechin; 21: epicatechin; 22: puerarin; 23: homoorientin; 24: vitexin; 25: isovitexin; 26: naringin; 27: procyanidine A_2_; 28: procyanidine B_2_; 29: 4-hydroxybenzaldehyde; 30: 6-gingerol; 31: vanillin.

Figure 4 shows chromatogram for 31 phenolic compounds standards. Table 3 showed that the phenolic compounds were identified and quantified by germination and USG treatment in the BHB (free fraction and bound fraction). In total, 27 free and 21 bound phenolic compounds were detected in the untreated sample. In addition, 26 free and 19 bound phenolic compounds were detected in germination, while 27 and 20 bound phenolic compounds were detected in USG. Compared with the untreated sample, germination reduced the types of free and bound phenolic compounds in BHB, while USG reduced only the types of bound phenolic compounds. However, both germination treatment and USG treatment significantly increased the total phenolic and free phenolic content of BHB, with the USG showing a higher enrichment effect. Total free phenolic, total bound phenolic, and total phenolic in USG were the highest, with 1.11, 1.31, and 1.17 times higher than those of germination extract, respectively. Among them, bound phenolic acids (242.06 ± 2.44 mg/100 g), free flavonoids (522.18 ± 8.54 mg/100 g), and total flavonoids (530.09 ± 2.73 mg/100 g) showed a marked increase among the primary phenolic substances, which were 1.44, 1.46, and 1.45 times relative to those in germination samples, respectively. Furthermore, USG decreased the total amount of free phenolic acids (80.25 ± 0.95 mg/100 g), bound flavonoids (7.95 ± 0.46 mg/100 g), total monomeric phenols (98.89 ± 1.55 mg/100 g), free monomeric phenols (64.97 ± 1.67 mg/100 g), and bound monomeric phenols (33.92 ± 0.87 mg/100 g) compared to the germination sample. It can be attributed to the mechanical and chemical effects of sound waves that destroy the structure of some phenolic compounds during the ultrasonic treatment [29], causing their decomposition to reduce their content.

In addition, the germination treatment had a significant increase in 83.33% of free phenolic acids, 33.33% of bound phenolic acids, 37.50% of free flavonoids, 25.00% of bound flavonoids, 75.00% of free monomeric phenols, and 80.00% of bound monomeric phenols, while the USG treatment had a significant increase in 66.67% of free phenolic acids, 33.33% of bound phenolic acids, 43.75% of free flavonoids, 55.56% of bound flavonoids, 60.00% of free monomeric phenols, and 40.00% of bound monomeric phenols. It indicates that the germination and USG have certain enrichment effects on the phenolic substances in BHB, among which the germination could effectively increase the free phenolic acids and monomeric phenols substances (free and bound forms) in BHB while the USG could more effectively increase the bound phenolic acids and free flavonoids. The total amount of phenolic substances in BHB was significantly higher in the USG sample (951.29 ± 2.62 mg/100 g DW) than in the germination (816.03 ± 3.38 mg/100 g DW).

It had been reported that the phenylalanine pathway was believed to be the most important and common metabolic pathway for synthesis of phenolic substances, and phenylalanine ammonia lyase (PAL), the major rate-limiting enzyme for synthesis of plant polyphenols, was generally activated by germination [30]. In addition, the recovery of relevant enzyme activity or synthesis of necessary enzymes after germination allows the release of phenolic substances bound to the cell wall, thus increasing phenolic and flavonoid contents. Therefore, germination can promote the release of phenolic substances, and this conclusion is consistent with the finding that the total phenolic content of BHB treated with germination increased by 24.70% compared with untreated samples in this paper. It had been reported that brown rice [31], oats [32], and canary seeds [33] after germination treatment show an increasing trend. However, the phenolic content was significantly higher using the USG treatment than the results of the germination treatment, probably because ultrasonic makes the seed shell and accelerates the hydration process [34,35], leading to changes in the molecular structure and catalysis of enzymes, triggering the defense reaction systems, and enhancing the production of secondary metabolites such as the polyphenols [36]. The cavitation and mechanical effects of ultrasonic enhance the permeability of cell membranes and promote diffusion and transmembrane transport of ions and metabolites [37]. According to Wang’s report [38], the flavonoid content of mung bean seeds was significantly increased after ultrasonic treatment and germination for 48 h and was significantly higher than that of the samples germinated alone, which is consistent with the results of this study.

Comparing the changes of different phenolic substances in BHB after germination and USG (Table 3), the main phenolic substances in germination sample, i.e., free protocatechuic acid, free *p*-hydroxybenzoic acid, free *p*-coumaric acid, free epicatechin, free 4-hydroxybenzaldehyde, bound 4-hydroxybenzaldehyde, free catechin, and free phlorogucinol increased remarkably, which were 14.51, 4.74, 11.49, 3.99, 2.71, 2.28, 1.81, and 1.61 times higher than those in the untreated group, respectively, among which protocatechuic acid, *p*-coumaric acid, catechin, and phlorogucinol were the main characteristic phenolic substances in germination, and their content accounted for 70.53% of the total free phenolic content. The contents of bound ferulic acid, free kaempferol, free luteolin, free diosmetin, and free catechin increased remarkably, which were 1.13, 68.92, 2.30, 2.10, and 2.08 times higher than those in the untreated group, respectively, among which ferulic acid, kaempferol, diosmetin, and catechin were the main characteristic phenolic substances in USG, and their content accounted for 65.48% of the total free phenolic content. The results indicated that germination and USG had certain selectivity for increasing the content of phenolic substances in BHB. Among them, catechin is a characteristic flavonoid in BHB, and its content can be significantly increased by germination and USG, and which is significantly higher than that of blue highland barley (37.91–47.98 mg/100 g DW) [39]. Unlike buckwheat where the main flavonoid is rutin (14.65–40.63 mg/100 g) and oats where the main flavonoid is quercetin (3.10–8.90 mg/100 g) [40], the catechin content can subsequently be used as a basis for evaluation of polyphenol-enriched product development in BHB. In addition, compared with the main phenolic substances in untreated samples, the bound geranin in the germination sample and bound diosmetin in the USG sample were not detected, which may be explained by the susceptibility of some phenolic monomers to oxidation and degradation during germination, or interactions and complexations between phenolic compounds [41]. Other phenolic substances were not analyzed in detail due to their relatively low content.

### 2.3. Effects of USG on the In Vitro Antioxidant Activity of BHB

The changes of antioxidant activity of BHB before and after USG are shown in Table 4. Relative to the untreated group, DPPH· scavenging ability, FRAP reducing power, and ABTS^+^ scavenging ability of BHB by germination and USG were significantly higher than those in the untreated sample. Compared to the untreated, the germination sample showed higher FRAP reduction power (4883.24 ± 12.56 umol/100 g DW), while the USG sample had higher DPPH· scavenging ability (6976.23 ± 47.19 umol/100 g DW), which increased by 51.77% and 21.55% higher, respectively, similar to findings of Tang’s study [26]. It used the synthetic index method to evaluate the antioxidant activity of BHB before and after treatment (Table 4). APC index from largest to smallest was USG sample (97.91%) > germination sample (97.27%) > untreated sample (76.52%). Therefore, USG was superior in improving the content of phenolic substances and their antioxidant activities in BHB.

### 2.4. Correlations between Antioxidant Capacity and Phenolic Compounds

In order to clarify the relationship between the contents of the free, bound, and total phenolic compounds and their antioxidant activities in BHB, the characteristic phenolic monomers of BHB under different treatments and the monomers with more significant changes between treatments were selected for correlation analysis, and the analysis results are shown in Table 5. TPC showed an extremely significant positive correlation with the DPPH· scavenging ability and ABTS^+^· scavenging ability and the FRAP reducing power (*p* < 0.01). This result is consistent with Boubakri’s study [42], which was a strong correlation between the content of total phenolic and DPPH· scavenging ability, ABTS^+^· scavenging ability, and FRAP reducing power of Tunisian barley. In addition, TFC showed a significant positive correlation with the FRAP and ABTS^+^· scavenging ability, in which catechin and isovitexin were the main contributors. Additionally, Zhao [43] also mentioned that DPPH· scavenging ability and ABTS^+^· scavenging ability were high correlations with TPC and some individual phenolic contents, especially with the amount of catechin.

In addition, free phenolic content and free flavonoid content were highly significantly and positively correlated with ABTS^+^· scavenging ability and FRAP reducing power (*p* < 0.01), but significantly and positively correlated with DPPH· scavenging ability (*p* < 0.05) or not. Free kaempferol, free catechin, and free homogentisic acid were highly significantly and positively correlated with DPPH· scavenging ability. However, the findings of this paper differ from the results reported by Abdel-Aal [44] that free *p*-coumaric acid has a significant positive correlation on DPPH· scavenging ability, which could result from different varieties studied, growth environments, and different phenolic enrichment methods. Free catechin, free isovitexin, free homogentisic acid, free *p*-hydroxybenzoic acid, free *p*-coumaric acid, and free 4-hydroxybenzaldehyde showed highly significant positive correlations with FRAP reducing power and ABTS^+^· scavenging ability. These results indicated that the content of free phenolic in BHB significantly related to their antioxidant capacity. Free kaempferol, free catechin, and free homogentisic acid were the main contributors to the DPPH· scavenging ability. Free catechin, free isovitexin, free homogentisic acid, free *p*-hydroxybenzoic acid, free *p*-coumaric acid, and free 4-hydroxybenzaldehyde were the major contributors to FRAP reducing power and ABTS^+^· scavenging ability. In contrast, free diosmin and procyanidin B_2_ were significantly and negatively correlated with FRAP reducing power and ABTS^+^· scavenging ability.

The content of bound phenolic compounds was highly significantly and positively correlated with DPPH· scavenging ability and FRAP reducing power. This means that the bound phenolic extract of BHB contains a greater number of phenolic substances scavenging DPPH-radicals and FRAP. Bound *p*-coumaric acid, bound homogentisic acid showed highly significant and significant positive correlation with DPPH· scavenging ability, and bound *p*-coumaric acid, bound 4-hydroxybenzaldehyde showed highly significant and significant positive correlations with FRAP reducing power. This implies that bound *p*-coumaric acid, bound homogentisic acid may be the main contributor to DPPH· scavenging ability and bound *p*-coumaric acid, bound 4-hydroxybenzaldehyde may be the main contributor to FRAP reducing power. This signifies that different kinds of monomer phenols exhibit selectivity for different antioxidant activity evaluation methods [45]. In the present study, the reason for the differences in the main contribution of monomeric polyphenols in the various antioxidant systems may have been that the composition and content of polyphenols differed due to the different species and growth environment, in addition to being closely related to the way in which polyphenols were enriched in this study. In the present study, it was found that the content of total phenolic compounds was positively correlated with all three antioxidant activities; therefore, the effective increase of TPC has some application value for improving the antioxidant activity of BHB.

## 3. Materials and Methods

### 3.1. Materials and Reagents

Kunlun 20, a highland barley variety bred by the Institute of Crop Breeding and Cultivation, Qinghai Academy of Agricultural and Forestry Sciences, was planted and cultivated at its experimental site (Xining, Qinghai) in 2019 (altitude of 2300 m; 36°67′ N 101°77′ E). The 31 kinds of polyphenol standards including 6 kinds of phenolic acids (homogentisic acid, vanillic acid, protocatechuic acid, *p*-hydroxybenzoic acid, *p*-coumaric acid, ferulic acid), 20 kinds of flavonoids (sesamol, kaempferol, luteolin, maltol, taxifolin, rutin, diosmin, kaempferol-3-O-rutinoside, myricetin, quercetin, diosmetin, catechin, epicatechin, puerarin, homoorientin, vitexin, isovitexin, naringin, procyanidine A_2_, procyanidine B_2_), and 5 kinds of monomeric phenols (phlorogucinol, pyrogallol, 4-hydroxybenzaldehyde, 6-gingerol, vanillin) with purity ≥ 98% were procured from Shanghai Yuanye Bio-Technology Co., Ltd. (Shanghai, China). Folin-Ciocalteu reagent (GR) was provided by Beijing Solarbio Science & Technology Co., Ltd. (Beijing, China). The 1,1-diphenyl-2-picrylhydrazylradical (DPPH), 2,4,6-tripyridyl-s-triazine (TPTZ), 2,20-azinobis-(3-ethylbenzthiazoline-6-sulfonate) (ABTS), and 6-hydroxy-2,5,7,8-tetramethylchroman-2-carboxylic acid (Trolox) with BR level were provided by Sigma Co. (St. Louis, MO, USA). All of the other chemicals and reagents used in the experiments were domestic analytical pure reagents.

### 3.2. Sample Preparation

Untreated sample: highland barley was crushed using a portable universal grinder (HK-04A, Shanghai Keheng Industrial Development Co., Ltd., Shanghai, China).

The germination sample: Highland barley was soaked in 1% sodium hypochlorite solution for 20 min and then drained and soaked for 12 h at 20 °C. Subsequently, the soaked barley was placed in Petri dishes lined with double-layer filter paper and germinated at 28 °C for 60 h in a humidified incubator (HWS-250F, Ningbo, Jiangsu, China), where distilled water was sprayed every 4 h to keep the seeds moist.

The USG sample: Highland barley was soaked in 1% sodium hypochlorite solution for 20 min and then drained and soaked for 12 h at 20 °C. According to ultrasonic power and ultrasonic time in the test, the barley was subjected to stress using an ultrasonic bath (KQ-5000DE, Kunshan, Jiangsu, China) and the temperature was held constant by circulating water through a water jacket (ordinary water flow: from 5 to 7 mL/s). After ultrasound pretreatment, the seeds germinated at 28 °C for certain hours under the same conditions as the germination sample. All of the samples were crushed and filtered through an 80-mesh sieve for subsequent use.

### 3.3. Research Procedures

#### 3.3.1. Extraction of Free and Bound Phenols

The extraction of free and bound phenolic compounds from BHB was performed according to the method of Jin [46]. Free phenolic extraction was performed as follows: to 25 mL of acetone solution (80% *v*/*v*), 1.0 g of whole barley powder was added, shaken, and ultrasonicated for 20 min at 20 °C, followed by centrifugation at 3000× *g* for 15 min (at 4 °C) (TGL-20M, Xiangyi, Changsha, China) to collect the supernatant. The residue was also treated as described above to collect the supernatant. After collecting the supernatant, the residue was subjected to the above treatment two times using the same method. The supernatant obtained in the three procedures were combined and spun at 45 °C using a rotary evaporator (Retavapor R-215, Buchi, Switzerland) to dry the mixture. The residue volume was made to 10 mL with anhydrous methanol and after conventional filtration (0.45 µm organic membrane filters) to obtain free phenol extract. This was stored at −20 °C in the dark.

Bound phenolic extraction was performed as follows: to the residue after the extraction of free phenols, 20 mL of n-hexane was added. The mixture was shaken and centrifuged at 3000 r/min for 5 min; the supernatant was discarded, following which, 17 mL of 11% hydrochloric acid-methanol solution was added to the precipitate, shaken well, and extracted twice using 20 mL of ethyl acetate following incubation in a water bath at 70 °C for 1 h. The extracts were mixed, rotor-evaporated at 45 °C, and fixed to 10 mL with anhydrous methanol after conventional filtration (0.45 µm organic membrane filters) to obtain barley-bound phenol extract. This was stored in the dark at −20 °C.

#### 3.3.2. Determination of Total Phenolic Contents (TPC) and Total Flavonoid Contents (TFC)

The TPC and TFC were measured following Yang’s method [39]. Polyphenol content determination was performed as follows: 125 µL of extraction was aspirated into a test tube, followed by the addition of 125 µL of foline-phenol and 500 µL of distilled water; the samples were shaken well. After incubation for 6 min, 1.25 mL of 7% Na_2_CO_3_ solution was added, followed by the addition of 1 mL of distilled water. After 1.5 h of incubation at room temperature (in dark), the absorbance was measured twice using a UV-vis spectrophotometer (N4S, Yidian, Shanghai, China) at 765 nm. All samples and measurements were performed in triplicates. Gallic acid was used as the standard, the TPC was calculated according to the standard curve (Y = 0.0042X + 0.0124 [0–300 µg/mL, R^2^ = 0.9996]), and the TPC was expressed as mg gallic acid equivalents (GAE)/100 g DW. Flavonoid content determination was as follows: 100 µL of extraction was aspirated into a test tube, followed by the addition of 200 µL of 5% NaNO_2_ solution; the samples were shaken well. After incubation for 6 min, 200 µL of 10% Al(NO_3_)_3_ solution was added and shaken well. After 6 min, 2 mL of 4% NaOH solution was added, followed by the addition of 2.5 mL of distilled water. After 15 min of incubation at room temperature (in dark), the absorbance was measured twice at 510 nm. All samples and measurements were performed in triplicates. The TFC was calculated according to the standard curve (Y = 0.0055X − 0.0047 [0–80 µg/mL, R^2^ = 0.9947]), and the TFC were expressed as mg rutin equivalents (GAE)/100 g DW.

#### 3.3.3. Determination of In Vitro Antioxidant Activity of Black Barley Polyphenol Extracts

The antioxidant capacities for the samples were determined using three different assays: FRAP reducing power, DPPH· scavenging ability, and ABTS^+^· scavenging ability. The antioxidant activity was determined following Yang’s method [39]. FRAP reducing power was determined as follows: The FRAP working solution was composed of 300 mmol/L pH 3.6 sodium acetate buffer solution, 20 mmol/L FeCl_3_ solution, and 10 mmol/L TPTZ solution (10:1:1, *v*/*v*/*v*). Typically, 1 mL of polyphenol extract and 4.5 mL of FRAP working solution were mixed in a test tube, and the absorbance was detected at 593 nm after 30 min of incubation in the dark. The FRAP reducing power was calculated according to the standard curve (Y = 0.0072X − 0.0012 [0–300 µmol/L, R^2^ = 0.9992]), and the results were expressed in µmol Trolox in 100 g of sample (dry weight). DPPH· scavenging ability was assessed as follows: 1 mL of polyphenol extract and 4 mL of 0.1 mmol/L DPPH· methanol solution were mixed in a test tube and the absorbance was detected at 517 nm after 30 min of incubation in the dark. The DPPH· scavenging ability of the extract was calculated according to the standard curve (Y = 0.0042X + 0.9163 [0–140 µmol/L, R^2^ = 0.9928]), and the results were expressed in µmol Trolox in 100 g of sample (dry weight). ABTS^+^· scavenging ability was estimated as follows: the ABTS^+^· working solution was prepared by mixing 5 mL 7 mmol/L ABTS solution with 88 µL 140 mmol/L potassium persulfate solution and was then kept in the dark for 12–16 h. The stock solution was diluted to a UV–Vis absorbance of 0.7 ± 0.02 using anhydrous methanol (1:100, *v*/*v*) before use. In most cases, 200 μL of polyphenol extract and 4 mL of the ABTS^+^· scavenging working solution were mixed in a test tube, and the absorbance was estimated at 734 nm after 30 min of incubation in the dark. The ABTS^+^· scavenging ability of the extract was calculated according to the standard curve (Y = −0.001X + 0.6242 [0–300 µmol/L, R^2^ = 0.9907]), and the results were expressed in µmol Trolox in 100 g of sample (dry weight).

#### 3.3.4. Response Surface Test

According to the findings of previous multivariate analyses, the Box–Behnken RSM was employed to design a test using germination time (X_1_), ultrasound power(X_2_), and ultrasound time (X_3_) as the significant influencing factors and TPC (Y_1_) and TFC (Y_2_) as the response values. The experimental design is presented in Table 6.

#### 3.3.5. Composition of Phenolic Substances

The composition of the phenolic compounds was determined based on the literature [47] using a Hypersil GOLD aQ column (100 mm × 2.1 mm) and the UPLC-Q-Orbitrap MS (Q-Exactive, Dionex Ultimate 3000 RSLC, ThermoFisher, USA). Mobile phase: 0.9% acetic acid-water solution (phase A) and methanol (phase B); flow rate, 0.3 mL/min; and sample size, 1 μL. Scan mode: Full MS; electrospray ionization (ESI); carrier gas, high-purity nitrogen (purity > 99.5%); spray voltage: 2.80 kV; capillary temperature: 300 °C; auxiliary gas heat source temperature: 300 °C, and positive/negative ion scanning mode. All samples and measurements were performed in triplicates.

#### 3.3.6. Antioxidant Potency Composite Index (APC index)

The antioxidant activity was compared in different treatments of BHB using the APC index method [48]. Antioxidant index score/% = sample score/best score × 100. The sample score means the sample measurement value of the method and the best score means the maximum value measured by this method. The average of all tests for each treatment was then taken for the antioxidant potency composite (APC) index.

#### 3.3.7. Statistical Analysis

Excel 2003 (Microsoft, Redmond, WA, USA) and SPSS 26 (SPSS Inc., Chicago, IL, USA) were used for data organization and analysis. Data were expressed as the mean ± standard deviation (mean ± SD) of the results of three experimental replicates. Duncan’s method was used for the analysis of the significance of the differences among multiple samples. Design Expert 10.0.1 software was used for central composition design.

## 4. Conclusions

In summary, Box–Behnken RSM was used to optimize the processing parameters for enriching polyphenols in BHB by USG. The optimal process parameters were obtained with ultrasonic pre-treatment power 350 W at 30 °C for 25 min, and a germination time of 64 h. Under these conditions, TPC, TFC, and APC index indices in BHB were increased significantly by 28.55%, 10.15%, and 27.95% respectively, compared to the untreated sample. HPLC–MS/MS analysis demonstrated that USG significantly increased total phenolic and antioxidant activity of BHB compared to germination, making it an effective method for enriching polyphenols. Germination could effectively enrich free phenolic acids and monomeric phenols, while USG could more effectively enrich bound phenolic acids and free flavonoids, where catechin is a characteristic substance in BHB, which was significantly increased in germination and USG, and which could subsequently be used as a basis for evaluation of polyphenol-enriched product development in BHB. Our findings may provide a reference for further improving the utilization of BHB.

## Figures and Tables

**Table 1:** Design and results of the Box–Behnken test.

Number	X_1_: Germination Time (h)	X_2_: Ultrasound Power (W)	X_3_: Ultrasound Time (min)	TPC (mg/100 g DW)	TFC (mg/100 g DW)
1	−1	−1	0	581.66 ± 3.69	46.06 ± 0.45
2	1	−1	0	600.74 ± 3.30	56.78 ± 0.31
3	−1	1	0	581.23 ± 1.17	47.69 ± 0.15
4	1	1	0	629.25 ± 3.36	56.70 ± 1.33
5	−1	0	−1	598.94 ± 0.97	49.28 ± 1.48
6	1	0	−1	649.46 ± 4.20	58.32 ± 0.56
7	−1	0	1	574.25 ± 4.87	45.34 ± 0.53
8	1	0	1	645.70 ± 4.02	57.60 ± 0.39
9	0	−1	−1	593.19 ± 2.33	55.17 ± 1.84
10	0	1	−1	630.20 ± 3.52	56.26 ± 0.81
11	0	−1	1	591.37 ± 7.78	53.75 ± 1.13
12	0	1	1	601.80 ± 4.56	53.27 ± 0.29
13	0	0	0	684.17 ± 6.11	58.47 ± 1.72
14	0	0	0	687.60 ± 2.09	59.12 ± 1.47
15	0	0	0	668.43 ± 8.78	59.17 ± 0.64
16	0	0	0	679.51 ± 3.37	58.63 ± 1.34
17	0	0	0	686.39 ± 9.54	58.88 ± 1.19

**Table 2:** Analysis of variance based on the regression model for TPC and TFC.

Source of Variation	TPC			TFC		
	F-Value	*p*-Value	Significance	F-Value	*p*-Value	Significance
Model	31.01	<0.0001	**	354.08	<0.0001	**
X_1_	46.95	0.0002	**	1852.18	<0.0001	**
X_2_	7.49	0.0290	*	5.13	0.0578	
X_3_	4.52	0.0711		90.51	<0.0001	**
X_1_ X_2_	2.20	0.1816		6.43	0.0389	*
X_1_ X_3_	1.15	0.3190		22.82	0.0020	**
X_2_ X_3_	1.86	0.2153		5.42	0.0527	
X_1_^2^	54.28	0.0002	**	754.47	<0.0001	**
X_2_^2^	101.82	<0.0001	**	238.06	<0.0001	**
X_3_^2^	37.48	0.0005	**	107.99	<0.0001	**
Lack of fit	2.33	0.2161		1.54	0.3343	
R^2^		0.9755			0.9978	
Adjusted R^2^ (R^2^_adj_)		0.9441			0.9950	

* and ** indicate a significant (*p* < 0.05) and a highly significant difference (*p* < 0.01), respectively.

**Table 3:** Composition and content of phenolic compounds in BHB before and after USG treatment (mg/100 g DW).

	Untreated Group			Germination Group			USG Group		
	Free	Bound	Total	Free	Bound	Total	Free	Bound	Total
**Flavonoids**									
Kaempferol	0.36 ± 0.05 ^b^	0.87 ± 0.14 ^b^	1.22 ± 0.19 ^b^	0.47 ± 0.02 ^b^	ND	0.47 ± 0.02 ^c^	24.81 ± 0.80 ^a^	2.40 ± 0.08 ^a^	27.22 ± 0.81 ^a^
Maltol	3.35 ± 1.31 ^a^	1.74 ± 0.11 ^b^	5.09 ± 1.38 ^a^	3.22 ± 0.94 ^a^	2.24 ± 0.24 ^b^	5.47 ± 0.61 ^a^	2.09 ± 0.33 ^b^	3.10 ± 0.54 ^a^	5.19 ± 0.28 ^a^
Taxifolin	0.29 ± 0.01 ^a^	0.58 ± 0.05 ^a^	0.87 ± 0.06 ^a^	0.21 ± 0.05 ^b^	0.50 ± 0.08 ^a^	0.70 ± 0.06 ^b^	0.15 ± 0.01 ^b^	0.30 ± 0.04 ^b^	0.45 ± 0.05 ^c^
Rutin	0.51 ± 0.03 ^a^	0.57 ± 0.02 ^a^	1.09 ± 0.03 ^a^	0.48 ± 0.05 ^a^	ND	0.48 ± 0.05 ^b^	0.47 ± 0.01 ^a^	ND	0.47 ± 0.01 ^b^
Diosmin	36.94 ± 0.84 ^a^	1.30 ± 0.27 ^a^	38.24 ± 1.11 ^a^	16.74 ± 0.48 ^c^	0.45 ± 0.24 ^b^	17.19 ± 0.36 ^c^	19.87 ± 1.27 ^b^	0.47 ± 0.01 ^b^	20.34 ± 1.26 ^b^
Kaempferol-3-O-rutinoside	0.42 ± 0.05 ^a^	ND	0.51 ± 0.06 ^b^	0.28 ± 0.03 ^b^	0.20 ± 0.10 ^a^	0.48 ± 0.09 ^b^	0.37 ± 0.03 ^a^	0.32 ± 0.05 ^a^	0.69 ± 0.03 ^a^
Myricetin	0.12 ± 0.01 ^b^	0.21 ± 0.04 ^a^	0.33 ± 0.02 ^a^	0.22 ± 0.01 ^a^	ND	0.22 ± 0.01 ^b^	0.13 ± 0.00 ^b^	ND	0.13 ± 0.00 ^c^
Quercetin	0.25 ± 0.05 ^a^	0.69 ± 0.05 ^a^	0.95 ± 0.04 ^a^	ND	0.19 ± 0.05 ^b^	0.19 ± 0.05 ^c^	0.13 ± 0.00 ^b^	0.26 ± 0.06 ^b^	0.38 ± 0.06 ^b^
Luteolin	5.70 ± 0.00 ^b^	ND	5.74 ± 0.01 ^b^	ND	ND	ND	13.11 ± 0.58 ^a^	ND	13.11 ± 0.58 ^a^
Diosmetin	60.07 ± 0.69 ^b^	5.52 ± 0.26 ^a^	65.58 ± 0.95 ^b^	26.53 ± 0.47 ^c^	4.89 ± 0.07 ^b^	31.42 ± 0.47 ^c^	125.98 ± 2.66 ^a^	ND	125.98 ± 2.66 ^a^
Catechin	132.59 ± 2.40 ^c^	ND	132.59 ± 2.40 ^c^	239.58 ± 4.05 ^b^	ND	239.58 ± 4.05 ^b^	276.06 ± 2.30 ^a^	ND	276.06 ± 2.30 ^a^
Epicatechin	4.58 ± 0.19 ^b^	ND	4.58 ± 0.24 ^b^	18.28 ± 0.50 ^a^	0.13 ± 0.09 ^a^	18.41 ± 0.44 ^a^	3.22 ± 0.03 ^c^	ND	3.22 ± 0.03 ^c^
Puerarin	ND	ND	ND	0.24 ± 0.11 ^a^	ND	0.24 ± 0.11 ^a^	ND	ND	ND
Homoorientin	3.72 ± 0.06 ^b^	0.59 ± 0.06 ^b^	4.30 ± 0.08 ^b^	1.40 ± 0.02 ^c^	0.54 ± 0.02 ^b^	1.94 ± 0.02 ^c^	4.12 ± 0.10 ^a^	0.82 ± 0.03 ^a^	4.95 ± 0.10 ^a^
Vitexin	0.46 ± 0.01 ^b^	ND	0.50 ± 0.02 ^a^	0.35 ± 0.01 ^c^	ND	0.35 ± 0.01 ^b^	0.48 ± 0.01 ^a^	ND	0.48 ± 0.01 ^a^
Naringin	ND	0.16 ± 0.10 ^a^	0.25 ± 0.10 ^a^	ND	ND	ND	ND	0.14 ± 0.07 ^a^	0.14 ± 0.07 ^a^
Isovitexin	4.12 ± 0.06 ^c^	ND	4.22 ± 0.06 ^c^	9.80 ± 0.03 ^a^	ND	9.80 ± 0.03 ^a^	7.49 ± 0.10 ^b^	0.14 ± 0.02 ^a^	7.62 ± 0.12 ^b^
Procyanidin A_2_	ND	ND	ND	0.32 ± 0.02 ^a^	ND	0.32 ± 0.02 ^a^	ND	ND	ND
Procyanidin B_2_	52.32 ± 0.74 ^a^	ND	52.32 ± 0.74 ^a^	41.05 ± 0.17 ^c^	ND	41.05 ± 0.17 ^c^	43.71 ± 1.06 ^b^	ND	43.71 ± 1.06 ^b^
Total flavonoids	305.79 ± 3.73 ^c^	12.23 ± 0.38 ^a^	318.09 ± 3.81 ^c^	357.62 ± 2.86 ^b^	9.13 ± 0.16 ^b^	366.32 ± 3.44 ^b^	522.14 ± 3.14 ^a^	7.95 ± 0.46 ^c^	530.09 ± 2.73 ^a^
**Phenolic acids**									
Ferulic acid	20.46 ± 0.34 ^a^	179.21 ± 5.56 ^b^	199.67 ± 5.25 ^a^	12.56 ± 0.06 ^b^	141.58 ± 1.97 ^c^	154.13 ± 1.97 ^b^	10.17 ± 0.78 ^b^	202.69 ± 1.85 ^a^	212.86 ± 2.05 ^a^
Protocatechuic acid	2.40 ± 0.06 ^b^	5.52 ± 0.09 ^b^	7.92 ± 0.10 ^b^	34.83 ± 1.49 ^a^	8.49 ± 0.48 ^a^	43.31 ± 1.01 ^a^	1.46 ± 0.21 ^b^	5.86 ± 0.07 ^b^	7.32 ± 0.18 ^b^
*p*-Hydroxybenzoic acid	2.87 ± 0.14 ^c^	1.29 ± 0.55 ^a^	4.17 ± 0.64 ^c^	13.61 ± 0.15 ^a^	1.28 ± 0.21 ^a^	14.88 ± 0.30 ^a^	8.35 ± 0.19 ^b^	0.94 ± 0.23 ^a^	9.39 ± 0.19 ^b^
Homogentisic acid	3.16 ± 0.10 ^c^	0.39 ± 0.20 ^b^	3.55 ± 0.29 ^c^	4.11 ± 0.18 ^b^	0.58 ± 0.49 ^b^	4.68 ± 0.65 ^b^	4.42 ± 0.15 ^a^	15.26 ± 0.49 ^a^	19.68 ± 0.60 ^a^
*p*-Coumaric acid	5.76 ± 0.39 ^c^	8.55 ± 0.22 ^c^	14.31 ± 0.25 ^c^	66.16 ± 0.36 ^a^	12.64 ± 0.41 ^b^	78.80 ± 0.58 ^a^	40.56 ± 0.70 ^b^	13.71 ± 0.37 ^a^	54.28 ± 0.71 ^b^
Vanillic acid	12.51 ± 0.41 ^c^	4.65 ± 0.45 ^a^	17.15 ± 0.26 ^b^	20.87 ± 1. 78 ^a^	3.53 ± 0.37 ^b^	24.39 ± 1.83 ^a^	15.30 ± 0.39 ^b^	3.60 ± 0.45 ^b^	18.90 ± 0.31 ^b^
Total phenolic acids	47.16 ± 1.05 ^c^	199.62 ± 11.42 ^b^	246.78.11 ± 5.14 ^b^	155.12 ± 3.17 ^a^	168.08 ± 1.04 ^c^	320.20 ± 3.29 ^a^	80.25 ± 0.95 ^b^	242.06 ± 2.44 ^a^	322.32 ± 2.53 ^a^
**Monomeric phenols**									
Phlorogucinol	51.34 ± 1.07 ^c^	9.78 ± 0.70 ^b^	61.12 ± 1.72 ^c^	82.73 ± 0.58 ^a^	13.41 ± 0.27 ^a^	96.14 ± 0.67 ^a^	57.55 ± 1.10 ^b^	9.29 ± 0.33 ^b^	66.85 ± 0.89 ^b^
Pyrogallol	0.16 ± 0.02 ^a^	2.60 ± 0.36 ^b^	2.76 ± 0.33 ^b^	ND	3.63 ± 0.29 ^a^	3.63 ± 0.29 ^a^	0.41 ± 0.18 ^a^	1.58 ± 0.15 ^c^	1.99 ± 0.24 ^c^
4-Hydroxybenzaldehyde	2.01 ± 0.11 ^c^	2.45 ± 0.17 ^c^	4.47 ± 0.14 ^c^	5.45 ± 0.04 ^a^	5.59 ± 0.08 ^a^	11.04 ± 0.06 ^a^	3.65 ± 0.46 ^b^	2.80 ± 0.15 ^b^	6.45 ± 0.31 ^b^
Sesamol	0.15 ± 0.02 ^b^	0.17 ± 0.09 ^a^	0.32 ± 0.11 ^a^	0.35 ± 0.12 ^a^	ND	0.35 ± 0.12 ^a^	ND	0.20 ± 0.04 ^a^	0.20 ± 0.04 ^b^
6-Gingerol	ND	ND	ND	ND	0.36 ± 0.00 ^a^	0.36 ± 0.00 ^b^	0.51 ± 0.06 ^a^	ND	0.51 ± 0.06 ^a^
Vanillin	4.53 ± 0.17 ^a^	16.34 ± 1.38 ^b^	20.87 ± 1.22 ^b^	1.90 ± 0.12 ^c^	16.08 ± 0.66 ^b^	17.99 ± 0.56 ^c^	2.85 ± 0.39 ^b^	20.05 ± 0.51 ^a^	22.90 ± 0.85 ^a^
Total monomeric phenols	58.20 ± 1.30 ^c^	31.33 ± 1.97 ^b^	89.53 ± 2.73 ^c^	90.43 ± 0.74 ^a^	39.08 ± 1.07 ^a^	129.51 ± 1.12 ^a^	64.97 ± 1.67 ^b^	33.92 ± 0.87 ^b^	98.89 ± 1.55 ^b^
Total phenolic acids+ total flavonoids+ total monomeric phenols	411.15 ± 4.14 ^c^	243.18 ± 4.75 ^b^	654.40 ± 2.08 ^c^	600.18 ± 1.93 ^b^	216.29 ± 2.18 ^c^	816.03 ± 3.38 ^b^	667.37 ± 4.08 ^a^	283.93 ± 2.34 ^a^	951.29 ± 2.62 ^a^

ND, not detected. Lower-case letters in the table indicate significant differences among different treatment of BHB in free, bound, and total fraction. (*p* < 0.05).

**Table 4:** Changes in total phenolic and in vitro antioxidant capacity in barley before and after USG treatment.

Group	FRAP Reducing Power (umol/100 g DW)	ABTS^+^· Scavenging Ability (umol/100 g DW)	DPPH·Scavenging Ability (umol/100 g DW)	APC Index (%)
Untreated group	3217.53 ± 30.82 ^c^	3663.47 ± 43.15 ^b^	5739.32 ± 28.23 ^c^	76.52 (3)
Germination group	4883.24 ± 12.56 ^a^	4501.00 ± 24.44 ^a^	6408.69 ± 73.11 ^b^	97.29 (2)
USG group	4632.18 ± 19.86 ^b^	4449.89 ± 42.47 ^a^	6976.23 ± 47.19 ^a^	97.91 (1)

Lower-case letters in the table indicate significant differences among different treatment of BHB in FRAP reducing power, and ABTS^+^ scavenging ability and DPPH· scavenging ability. (*p* < 0.05).

**Table 5:** Pearson correlation coefficients between antioxidant capacity and phenolic compounds.

	Free Phenolic Exaction			Bound Phenolic Exaction			Total Phenols		
	DPPH	FRAP	ABTS	DPPH	FRAP	ABTS	DPPH	FRAP	ABTS
Phenolic content	0.759 *	0.974 **	0.979 **	0.906 **	0.963 **	−0.278	0.935 **	0.965 **	0.981 **
Flavonoid content	0.137	0.863 **	0.845 **	0.114	0.642	0.305	0.304	0.794 *	0.743 *
Kaempferol	0.959 **	0.428	0.463	0.503	−0.068	−0.445	0.825 **	0.351	0.43
Diosmin	−0.548	−0.996 **	−0.987 **	−0.828 **	−0.912 **	−0.011	−0.809 **	−0.998 **	−0.989 **
Catechin	0.817 **	0.945 **	0.952 **				0.969 **	0.925 **	0.949 **
Isovitexin	0.321	0.943 **	0.930 **				0.646	0.968 **	0.942 **
Procyanidin B_2_	−0.485	−0.983 **	−0.978 **				−0.754 *	−0.989 **	−0.979 **
Homogentisic acid	0.827 **	0.919 **	0.941 **	0.783 *	0.302	-0.449	0.868 **	0.431	0.506
*p*-Hydroxybenzoic acid	0.228	0.908 **	0.890 **	−0.405	−0.189	0.078	0.512	0.914 **	0.873 **
*p*-Coumaric acid	0.299	0.938 **	0.922 **	0.977 **	0.904 **	−0.337	0.649	0.970 **	0.942 **
4-Hydroxybenzaldehyde	0.212	0.877 **	0.869 **	0.249	0.746*	0.154	0.34	0.818 **	0.766 *

Note: * and ** indicate a significant (*p* < 0.05) and a highly significant difference (*p* < 0.01), respectively. Phenolic content and flavonoid content detected by chemical method, respectively.

**Table 6:** Factors and levels in the response surface test.

Level	X_1_: Germination Time (h)	X_2_: Ultrasound Power (W)	X_3_: Ultrasound Time (min)
−1	48	300	20
0	60	350	25
1	72	400	30

## Data Availability

The data presented in this study are available within the article.

## Sample Availability

Samples of the compounds are not available from the authors.
